# Redox-sensitive and inflammatory pathway modulation by Chrysin confers protection against aflatoxin B1–induced hepato-renal damage

**DOI:** 10.1007/s12550-026-00655-z

**Published:** 2026-06-11

**Authors:** Esra Zeybek, Asım Kart, Özlem Özmen

**Affiliations:** 1 Department of Pharmacology and Toxicology, Faculty of Veterinary Medicine, Burdur Mehmet Akif Ersoy University, Burdur, Türkiye; 2 Department of Pathology, Faculty of Veterinary Medicine, Burdur Mehmet Akif Ersoy University, Burdur, Türkiye

**Keywords:** Aflatoxin B_1_, Chrysin, Oxidative stress, Sirtuin 1, Sialic acid

## Abstract

This study investigated the protective mechanisms of Chrysin (Ch) against aflatoxin B_1_ (AFB_1_)–induced hepato-renal toxicity in rats, focusing on oxidative stress, Sirtuin-1 (Sirt-1)/NF-κB signaling, apoptosis, and tissue remodeling pathways. Forty male Wistar albino rats were randomly allocated to Control, Ch, AFB_1_, and Ch+AFB_1_ groups. Chrysin (25 mg/kg BW /day) was administered orally for 11 days, while AFB_1_ (0.5 mg/kg BW /day) for 7 days. AFB_1_ exposure induced pronounced hepato-renal injury and histopathological alterations. At the molecular level, it caused excessive oxidative stress, evidenced by glutathione depletion and elevated malondialdehyde and 8-hydroxy-2′-deoxyguanosine levels, indicating lipid peroxidation and oxidative DNA damage. This redox imbalance activated inflammatory signaling, reflected by increased NF-κB p65 and TNF-α, alongside dysregulated Sirt-1 expression, suggesting disruption of redox-sensitive transcriptional control. Persistent oxidative and inflammatory stress further promoted mitochondrial apoptosis, as indicated by upregulated Caspase-3 and Caspase-9, and triggered compensatory proliferation, demonstrated by elevated PCNA and Ki-67 levels. Co-administration of Chrysin mitigated these biochemical and molecular alterations by restoring antioxidant defenses, suppressing NF-κB–mediated inflammation via modulation of the Sirt-1/NF-κB axis, and attenuating apoptotic and proliferative signaling. These molecular improvements were accompanied by preservation of hepatic and renal histoarchitecture. Although the animals exhibited subclinical biochemical and histological stress, no overt clinical illness was observed during the study. In conclusion, AFB_1_-induced hepato-renal toxicity is driven by interconnected oxidative, inflammatory, and apoptotic mechanisms. Chrysin provides multi-level protection by modulating these pathways, highlighting its potential as a protective agent against mycotoxin-induced toxic injury.

## Introduction

Mycotoxins are low-molecular-weight, chemically diverse secondary metabolites produced by various fungi, including *Aspergillus*, *Claviceps*, *Alternaria*, *Penicillium*, and *Fusarium* species (Gümüş et al. 2018). These compounds are ubiquitous in nature and pose serious risks to human and animal health due to their teratogenic, nephrotoxic, hepatotoxic, neurotoxic, mutagenic, and immunosuppressive effects. Among them, aflatoxins (AFs) represent the most significant group (Richard 2007). *Aspergillus flavus* and *Aspergillus parasiticus*, are particularly concerning because of their high prevalence, potent toxicity, and ability to enter the food chain from farm animals to humans via milk, eggs, and tissues, resulting in global public health challenges and substantial economic losses (Preetha et al. 2006).

Aflatoxin B_1_, classified as a Group 1 human carcinogen by the International Agency for Cancer Research (IARC), is frequently detected in cereals, oilseeds, and spices (Kos et al. 2020). Over 20 naturally occurring aflatoxin derivatives have been identified, including AFB_1_, AFB_2_, AFG_1_, AFG_2_, and AFM_1_. Among these, AFB_1_ is the most prevalent and hepatocarcinogenic. Acute ingestion of AFB_1_ can induce severe hepatotoxicity and hepatocellular necrosis, whereas chronic low-dose exposure leads to growth retardation, developmental abnormalities, and immune suppression (Jin et al. 2021).

The toxic and carcinogenic potential of AFB_1_ is primarily linked to its biotransformation in the liver, the main metabolic organ and primary target of the toxin. In hepatocytes, AFB_1_is converted into the highly reactive intermediate AFB_1_-8,9-epoxide by cytochrome P450 and aryl hydrocarbon hydroxylase enzymes. Under normal physiological conditions, these electrophilic epoxides are detoxified through conjugation with reduced glutathione (GSH), catalyzed by the glutathione S-transferase (GST). However, during high-dose or prolonged exposure, these detoxification mechanisms are overwhelmed. The accumulated reactive metabolites covalently bind to the N7 position of guanine residues in the DNA, inducing mutations and contributing to carcinogenesis (Lin et al. 2014). In parallel, AFB_1_ metabolism promotes excessive production of reactive oxygen species (ROS), including superoxide anion (O2-), hydroxyl radical (OH), and hydrogen peroxide (H_2_O_2_) triggering oxidative stress that damages lipids, proteins, and DNA, ultimately leading to hepatocyte apoptosis, autophagy, and necrosis (Liu et al. 2021).

To counteract AFB_1_-induced oxidative and toxic damage, phytochemicals with antioxidant properties have emerged as promising therapeutic agents. Flavonoids, a class of polyphenolic compounds abundant in plants, are central to this strategy. Chrysin (5,7-dihydroxyflavone) is a naturally occurring flavonoid found in high concentrations in honey, propolis, passionflower (*Passiflora caerulea*), and edible mushrooms such as *Pleurotus ostreatus* (Mani and Natesan 2018; Naz et al. 2019). Structurally, Chrysin, possesses a 15-carbon skeleton, comprising two benzene rings (A, B), and a heterocyclic xygen-containing ring (C), derives with the biological activity largely attributed to the double bond in ring C and the hydroxyl groups on rings B and C (Harris et al. 2006). Chrysin exhibits strong ROS scavenging activity, reduces lipid peroxidation, and enhances endogenous antioxidant enzyme activities, including catalase (CAT), superoxide dismutase (SOD), and glutathione peroxidase (GPx) (Farkhondeh et al. 2019). Additionally, Chrysin can induce apoptosis in cancer cells via caspase-3 activation highlights its potential as a chemopreventive and therapeutic agent (Khoo et al. 2010).

In recent years, the Sirtuin protein family, particularly Sirtuin-1 (Sirt-1) has attracted attention for its role in cellular homeostasis, gene regulation, and tumorigenesis. Concentrated in metabolically active tissues such as the liver, kidney, and brain, Sirt-1 regulates chromatin structure and gene expression, and its modulation is critical in understanding tissue responses to toxic and carcinogenic insults (Alcaín and Villalba 2009; Nogueiras et al. 2012).

Another important molecular marker of carcinogesis is sialic acid (N-acetyl-neuronamic acid-NANA), which participates in cell-cell recognition, immune signaling, and tumor progression. Changes in Sirt-1 and sialic acid levels thus provide valuable insights into the carcinogenic potential of AFB_1_ exposure. Sialic acid, a component of glycoproteins and glycolipids, participates in cellular recognition, communication, and immune signaling. While elevated levels have been associated with tumor progression in long-term studies, in the context of this subchronic AFB_1_ exposure, sialic acid serves as a sensitive molecular marker reflecting early hepatic alterations rather than overt malignancy (Adak et al. 2013; Schauer and Kamerling 2018).

This research aimed to investigate the protective efficacy of Chrysin against AFB_1_-induced hepato-renal toxicity, using molecular, biochemical, histopathological, and immunohistochemical analyses. Emphasis was placed on assessing both the toxic impact of AFB_1_ and the ameliorative benefits of Chrysin, rather than solely focusing on carcinogenesis, given the exposure duration and endpoints examined. Chrysin’s protective effects were evaluated through biochemical markers (oxidative stress: MDA, GSH; DNA damage: 8-OHdG), molecular indicators of cellular stress (Sirt-1 and sialic acid), and histopathological/immunohistochemical parameters, including inflammation, apoptosis, and cell proliferation, highlighting its capacity to counteract early molecular and cellular alterations induced by AFB_1_.

## Materials and methods

### Chemicals

Aflatoxins B_1_ (Cayman-11293) was obtained from Cayman, chrysin (Ch) (BCM-805131-1G) was obtained from Bostonchem, glutathione (GSH) (BLS-8577Ra), malondialdehyde (MDA) (BLS-8612 Ra), 8-hydroxydeoxyguanosine (8-OHdG) (BLS-8994Ra), tumor necrosis factor-alpha (TNF-α) (BLS-1396Ra), nuclear factor kappa B-p65 (NFκB-p65) (BLS-1693Ra) kits were obtained from Bostonchem, NAD-dependent Protein deacetylase Sirtuin-1 (Sirt 1) (E1145Ra) kit were obtained from BT LAB. N-Acetylneuraminic acid (Sigma-Aldrich 002076496), Hydrochloric acid (Merck 1.00314.2500), Perchloric acid (Merck 1.09065.1000), P-dimethylaminobenzaldehyde (Sigma-Aldrich 101785787).

### Animals

The rats used as animal material in the study were obtained from the Burdur Mehmet Akif Ersoy University Experimental Animal Production and Experimental Research Center. A total of 40 male Wistar Albino rats, weighing approximately 150–180 g and aged 8–10 weeks, were used in the study, with 10 rats in each group. The animals were kept in adjustable rooms with 12-hour light/dark cycles under appropriate humidity (55–65% humidity) and temperature (21–23 °C) conditions. All animals were provided with ad libitum pellet feed and water. Throughout the experiment, the animals were fed rat pellet feed containing 24% crude protein, 6.19% crude cellulose, and 3,100 kcal/kg metabolic energy. All procedures applied to the animals in the study were carried out in accordance with the approval of the Burdur Mehmet Akif Ersoy University Local Ethics Committee for Animal Experiments (16.03.2022/866).

### Experimental procedure

In this study, a total of 4 groups were formed: control (C), Chrysin (Ch), Aflatoxin B_1_ (AFB_1_), and a combination of Chrysin and Aflatoxin B_1_ (Ch+AFB_1_). The AFB_1_ dose to be given to the animals was determined using the research of Karaca et al. (2021), and the Chrysin dose was determined using the research of Pushpavalli et al. (2010). All substances were administered starting on the first day of the experiment. The control group was given DMSO (0.5 mg/kg BW/day orally) for 11 days. The Chrysin group was given 25 mg/kg BW/day chrysin for 11 days. The Aflatoxin B_1_ group was administered 0.5 mg/kg BW/day AFB_1_ via gavage for 7 days. In the group receiving both Chrysin and Aflatoxin B_1_, Chrysin was administered at a dose of 25 mg/kg BW/day for 11 days, starting on day 1 of the experiment. AFB_1_ was administered orally via gavage at a dose of 0.5 mg/kg BW/day from day 2 until day 8. A two-day break was given to observe the effects of the drug administration, and the study was terminated on day 14. All animals were anesthetized with ketamine (90 mg/kg BW/ intraperitoneal) and xylazine (10 mg/kg BW/ intraperitoneal), blood sampling was performed from the heart, and the animals were euthanized by cervical dislocation while under anesthesia. Blood and tissue (liver and kidney) samples were collected. All animals contributed tissue samples to both analytical workflows; specifically, each harvested organ was divided into two equal portions, with one half allocated to toxicological analysis and the other half processed for histopathological and immunohistochemical evaluations, ensuring that all animals were represented in all assessments. Experimental groups were designed as follows: **Control group**: Dimethyl sulfoxide (DMSO) 0.5 mg/kg BW/day by oral gavage for 11 days; **Ch group**: 25 mg/kg BW/day Chrysin (5% DMSO) by oral gavage for 11 days; **AFB**_**1**_ **group**: 0.5 mg/kg BW/day AFB_1_ (5% DMSO) by oral gavage for 7 days; **Ch+AFB**_**1**_ **group**: 25 mg/kg BW/day Chrysin + 0.5 mg/kg BW/day AFB_1_ by oral gavage (Chrysin was started one day before AFB_1_ administration and continued for three more days after AFB_1_ administration). 7 days AFB_1_ + 11 days Chrysin.

### Evaluation of serum biochemical parameters

Blood samples were collected into serum (anticoagulant-free) tubes and centrifuged at 2860xg for 10 min to separate the serum. *Aspartate aminotransferase* (AST), *alanine aminotransferase* (ALT), *gamma-glutamyl transferase* (GGT) activities were measured using an autoanalyzer (Gesan chem 200, Gesan Production srl, Campobello, Italy) with the relevant chemical kits following protocols optimized according to IFCC (International Federation of Clinical Chemistry and Laboratory Medicine) guidelines. Creatinine, and blood urea nitrogen (BUN) levels were determined using the Jaffè method adapted for kinetic measurement with ultraviolet (UV) detection.

### Homogenization of liver and kidney tissues and preparation of homogenate

Liver and kidney tissues were first washed with 0.9% saline solution and then homogenized in 0.01 M phosphate-buffered saline (pH: 7.4) at a ratio of 1 g tissue/9 ml buffer (1:10 dilution), in accordance with kit instructions. The homogenates were centrifuged at 41.500 x g for 45 min at 4 °C to obtain the supernatants, which were stored at -20 °C until analysis. Tissue homogenates were used to measure GSH, MDA, TNF-α, NFκB-p65, 8-OHdG, and Sirtuin-1 levels.

### Determination of total protein concentration

Total protein concentrations in tissue homogenates were determined using the Biuret method described by Gornall et al. (1949).

### Total sialic acid (TSA) analysis

Total sialic acid levels in serum were measurement according to the method reported by Sydow (1985) with slight modifications. Briefly, 0.2 mL of serum was mixed with 1.5 mL of perchloric acid, boiled in a water bath at 100 °C for 5 min, cooled to 4 °C, and centrifuged at 2500 x g for 4 min. A 1 mL aliquot of the supernatant was transferred to a clean test tube, mixed with 0.2 mL of Ehrlich reagent, and boiled at 100 °C for 15 min. After cooling, 1 mL of distilled water was added, and optical densities (OD) were read at 525 nm. Serum TSA levels were calculated using a standard curve prepared from serial dilution of N-acetyl neurominic acid (NANA; Sigma-Aldrich, St. Louis, MO, USA).

### Histopathological method

Liver and kidney tissue samples obtained during necropsy were fixed in 10% neutral buffered formalinfor 48 h. Following fixation, tissues were processed using a routine automated tissue processor (Leica ASP300S, Leica Microsystems, Wetzlar, Germany) and embedded in paraffin wax. After solidification, paraffin blocks were sectioned at a thickness of 5 μm using a rotary microtome (Leica RM2155, Leica Microsystems, Wetzlar, Germany). The sections were mounted on glass slides, deparaffinized, and stained with hematoxylin and eosin (H&E) for general histopathological evaluation. All histopathological evaluations were performed by an independent, board-certified veterinary pathologist who was blinded to the experimental groups and treatment allocations throughout the study, in order to minimize observer bias and ensure objectivity. Tissue sections were systematically examined under a light microscope, and representative histomorphological alterations in liver and kidney tissues were documented using a digital imaging system.

### Immunohistochemical method

For immunohistochemical analysis, 5 μm sections were mounted on poly-L-lysine–coated slides. Following deparaffinization and rehydration, endogenous peroxidase activity was blocked using 3% hydrogen peroxide (H₂O₂). Antigen retrieval was performed in citrate buffer using microwave heating. To reduce nonspecific binding, sections were incubated with normal serum prior to overnight incubation at + 4 °C with the following primary antibodies: caspase-3 (CASP3 Polyclonal Antibody [E-AB-13815], Elabscience, USA), caspase-9 (CASP9 Polyclonal Antibody [E-AB-30760], Elabscience, USA), PCNA (clone PC10, 307902; BioLegend, USA), and Ki-67 (sc-23900; Santa Cruz Biotechnology, USA), all diluted at 1:100 according to the manufacturers’ instructions. After washing with phosphate-buffered saline (PBS), sections were incubated with a Mouse and Rabbit Specific HRP/DAB Detection Kit (Micropolymer ab236466), and immunoreactivity was visualized using 3,3′-diaminobenzidine (DAB) as the chromogen. Sections were counterstained with Harris hematoxylin, dehydrated, and mounted. Negative control sections were processed by replacing the primary antibodies with antibody diluent.

Immunohistochemical evaluations were also performed by the same independent, board-certified veterinary pathologist under blinded conditions. Quantitative assessment was conducted by counting a total of 100 cells per Sect.  (20 cells in each of five randomly selected high-power fields at 40× magnification) in both liver and kidney tissues. Only cells exhibiting distinct brown cytoplasmic or nuclear staining were considered positive. The percentage of positive cells for caspase-3, caspase-9, PCNA, and Ki-67 was calculated using ImageJ software (version 1.48, NIH, USA). Representative photomicrographs were obtained using an Olympus CX41 light microscope equipped with a digital imaging software.

### Statistical analysis

The data obtained from the study were evaluated using SPSS 22 software. Results were expressed as arithmetic mean ± standard error (SE). The parametric-nonparametric nature of the data was primarily analyzed using the Shapiro-Wilk test. Since biochemical results, GSH, MDA, 8OHdG, TNF-α, NFκB-p65, and Sirt-1 levels showed a normal distribution, One-Way ANOVA was used, followed by Tukey test for pairwise comparisons between groups (post hoc tests). Values below *p* < 0.05 were considered statistically significant. Histopathological scores and the number of immunohistochemically positive stained cells were expressed as arithmetic mean ± standard error (SE). Since pathological scores did not show a normal distribution, the Kruskal-Wallis test and the Mann-Whitney U test with Bonferroni correction were used. Values of “p < 0.05” were considered statistically significant.

## Results

### The effect of Chrysin on biochemical parameters

**Fig. 1:**
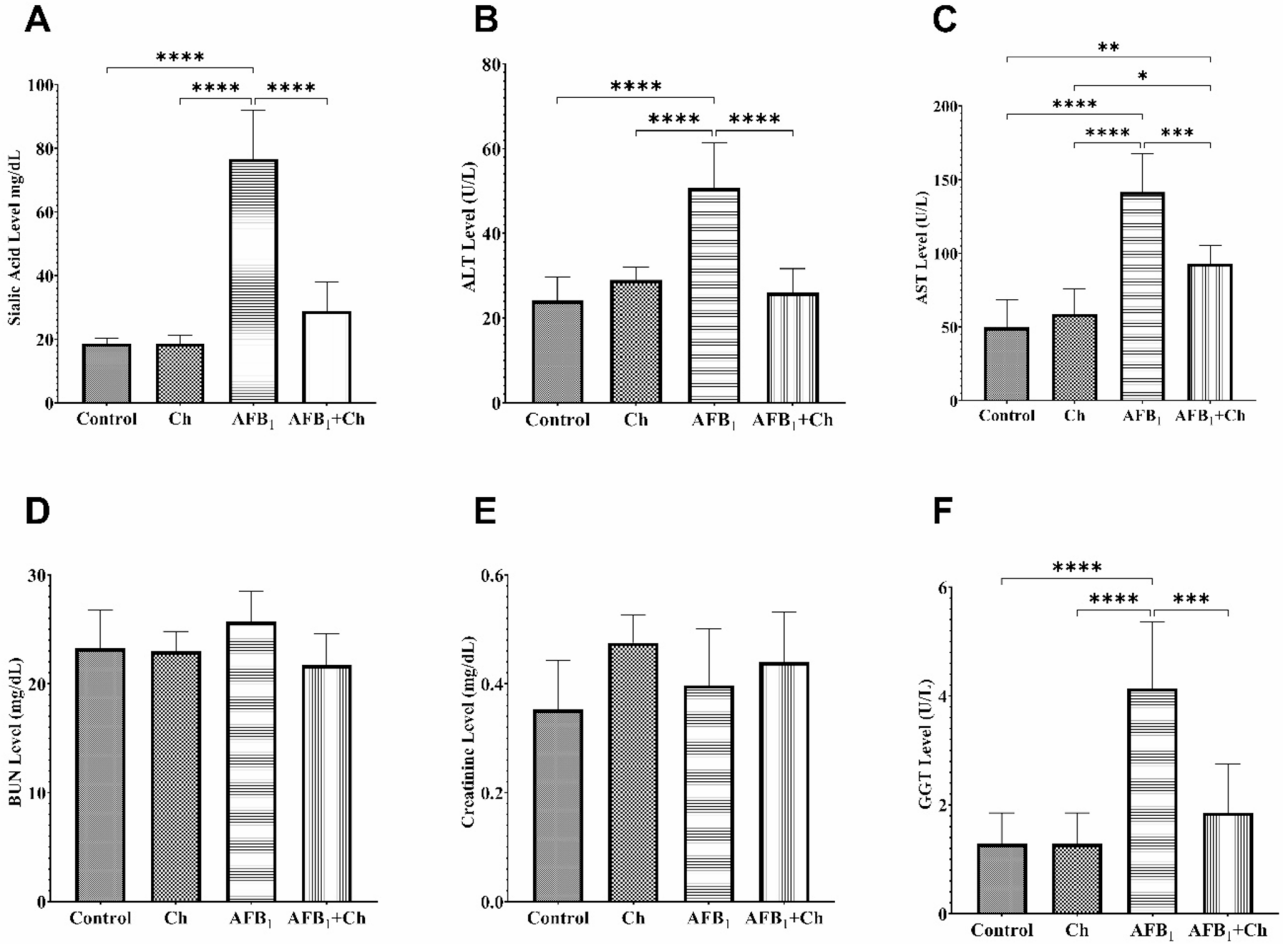
Serum total sialic acid (**A**), AST (**B**), ALT (**C**), GGT (**D**), BUN (**E**) and creatinine (**F**) levels of the study groups. Data are presented as mean ± standard error. Statistically significant differences are indicated by lines (*: *p* < 0.05). Groups without lines between them do not have a statistically significant difference, *n* = 10

A significant increase in serum AST, ALT, and GGT values was detected in aflatoxin B_1_ group compared to the control group (*p* < 0.05) (Fig. 1), while no statistically significant difference was found in creatinine and BUN values (*p* > 0.05) (Fig. 1). AST, ALT, and GGT values were significantly lower in AFB_1_+Ch group than in aflatoxin B_1_ alone (*p* < 0.05).

### The effect of Chrysin on antioxidant-oxidative stress parameters

**Fig. 2:**
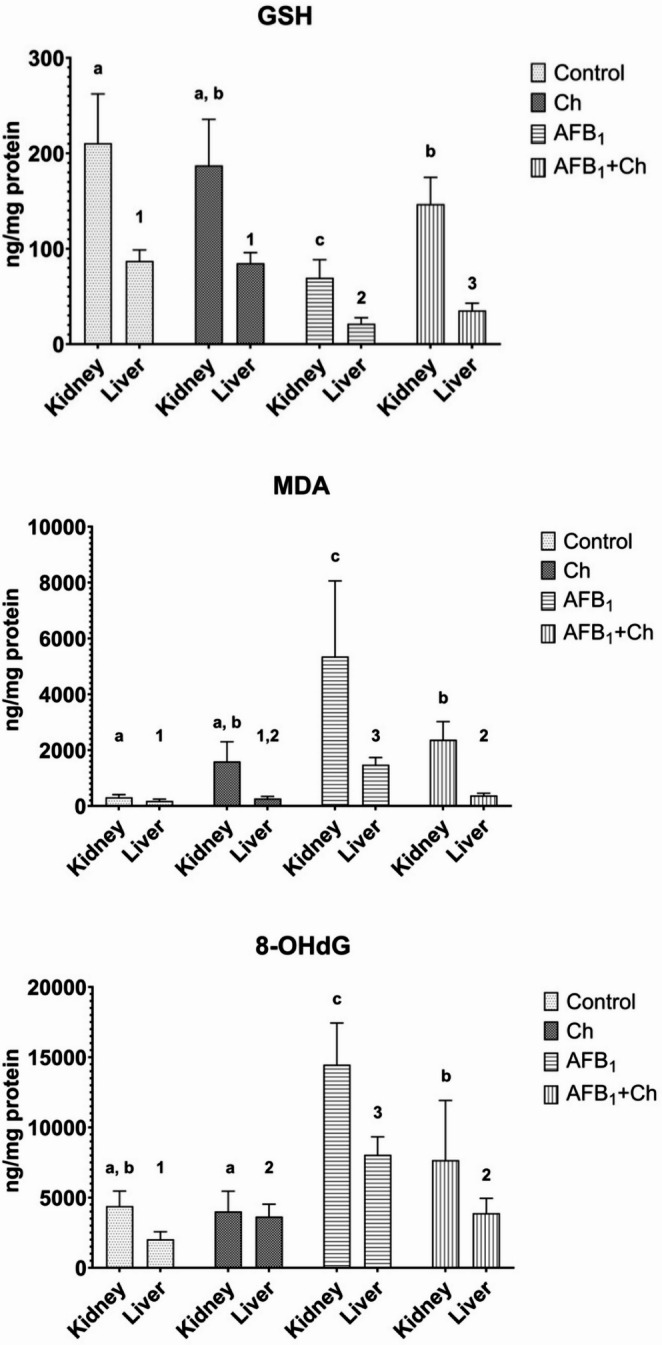
GSH, MDA, and 8-OHdG parameters in the liver and kidney tissues. Data are presented as mean ± standard error. Statistically significant differences are indicated by different letters in the kidney and different numericals in the liver (*: *p* < 0.05)., *n* = 10

In the group given aflatoxin B_1_, a significant decrease in GSH levels (*p* < 0.05) and a significant increase in MDA and 8-OHdG levels (*p* < 0.05) were detected in liver and kidney tissue samples when compared to the control group. In the group given chrysin and AFB_1_ together, a significant decrease in MDA and 8-OHdG levels (*p* < 0.05) and a significant increase in GSH levels (*p* < 0.05) were observed in comparison to AFB_1_ group (Fig. 2).

### The effect of Chrysin on inflammatory parameters and transcription factor parameters

**Fig. 3:**
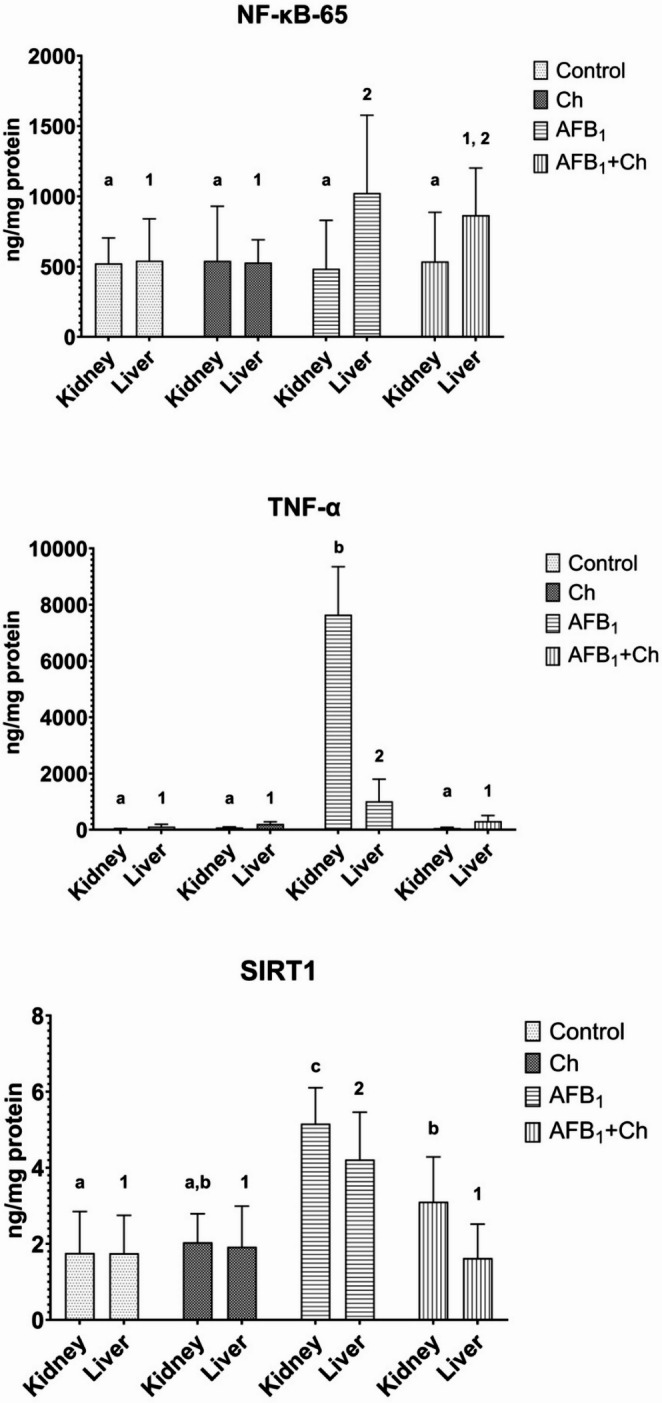
NFκB-p65, TNF-α, and Sirt-1 parameters in the liver and kidney tissues. Data are presented as mean ± standard error. Statistically significant differences are indicated by different letters in the kidney and different numericals in the liver (*: *p* < 0.05)., *n* = 10

In liver tissue samples, the aflatoxin B_1_-treated group showed a significant increase (*p* < 0.05) in NFκB-p65, TNF-α, and Sirt-1 levels compared to the control group. In kidney tissue, a significant increase was observed in TNF-α and Sirt-1 levels (*p* < 0.05), while no significant difference was found in NFκB-p65 levels (*p* > 0.05). The increased NFκB-p65, TNF-α, and Sirt-1 levels observed with aflatoxin B_1_ alone showed a significant decrease (*p* < 0.05) in AFB_1_+Ch group (Fig. 3).

### Serum total sialic acid levels in experimental groups

When serum sialic acid levels were compared between the aflatoxin B_1_ group and the control group, a significant increase was observed in the AFB_1_ group compared to the control (*p* < 0.05). A significant difference was found in sialic acid levels when comparing the chrysin group and the AFB_1_ group (*p* < 0.05). In the group where chrysin and AFB_1_ were administered together, a significant decrease in sialic acid levels was observed compared to the group receiving AFB_1_ alone (*p* < 0.05) (Fig. 1).

### Histopathological examination findings

**Fig. 4:**
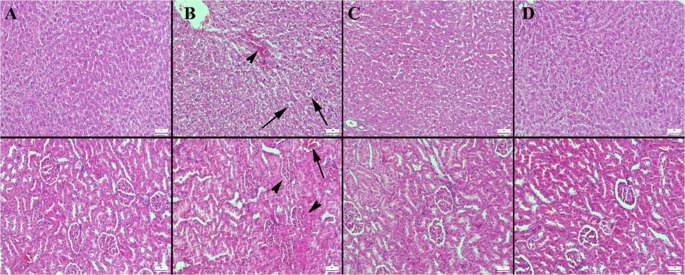
Histopathological appearance of liver (upper row) and kidney (lower row) tissues from the experimental groups. (**A**) Control group showing normal histological architecture. (**B**) AFB_1_ group exhibiting hepatic hemorrhage (arrowhead), severe hepatocellular steatosis and necrosis (arrows), along with marked renal hyperemia (arrow) and glomerulosclerosis (arrowheads). (**C**) AFB_1_+Ch group demonstrating a pronounced improvement in histopathological alterations. (**D**) Ch group showing normal liver tissue histology, H&E staining; scale bars = 50 μm and x400, *n* = 10

Histopathological examination revealed that rats in the control and Ch groups exhibited normal hepatic and renal architectures, characterized by well-preserved hepatocyte cords, intact sinusoids, normal glomerular structures, and well-organized renal tubules, with no apparent pathological alterations. In contrast, tissues from the AFB_1_ group showed pronounced pathological changes in both organs. Hepatic sections demonstrated severe hyperemia, focal microhemorrhages, marked Kupffer cell proliferation, and evident hepatic steatosis, accompanied by hepatocellular degeneration and necrosis; additionally, megalokaryosis and binucleated hepatocytes were observed in some animals, indicating cellular stress and regenerative activity. Renal sections from the same group exhibited hyperemia with occasional hemorrhagic foci, along with glomerular shrinkage and glomerulosclerosis, as well as tubular dilatation, epithelial desquamation, and megalokaryotic cells. Importantly, Ch treatment markedly ameliorated AFB_1_-induced histopathological damage in both the liver and kidney, as evidenced by a substantial reduction in vascular congestion, cellular degeneration, necrosis, and structural alterations, resulting in near-normal glomerular and tubular morphology in the AFB_1_+Ch group (Fig. 4).

### Immunohistochemical findings

**Fig. 5:**
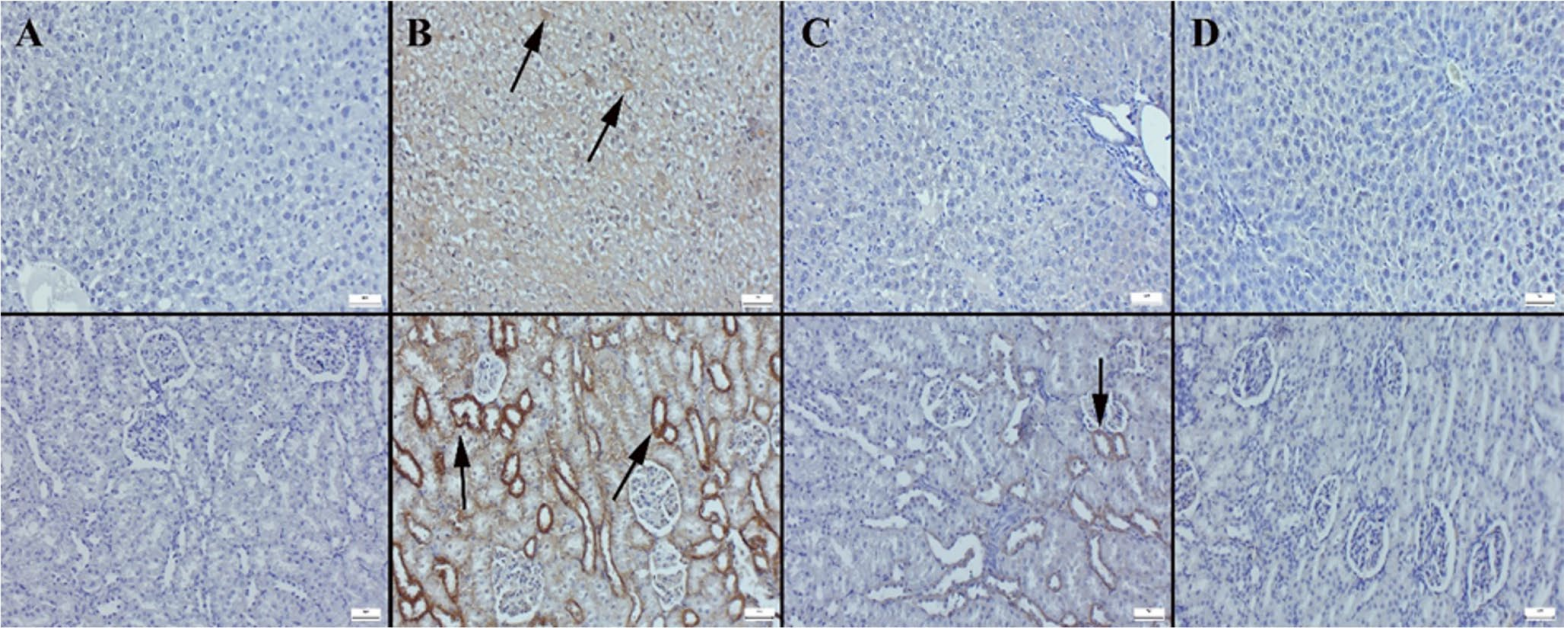
Immunohistochemical localization of caspase-3 expression in liver (upper row) and kidney (lower row) tissues from the experimental groups. (**A**) Control group showing negative caspase-3 immunoreactivity. (**B**) AFB_1_ group exhibiting markedly increased caspase-3 expression in hepatic hepatocytes and renal tubular epithelial cells (arrows). (**C**) AFB_1_+Ch group demonstrating a pronounced reduction in caspase-3 immunoreactivity. (**D**) Ch group showing negative caspase-3 expression. Streptavidin–biotin peroxidase method; scale bars = 50 μm and x400, *n* = 10

**Fig. 6:**
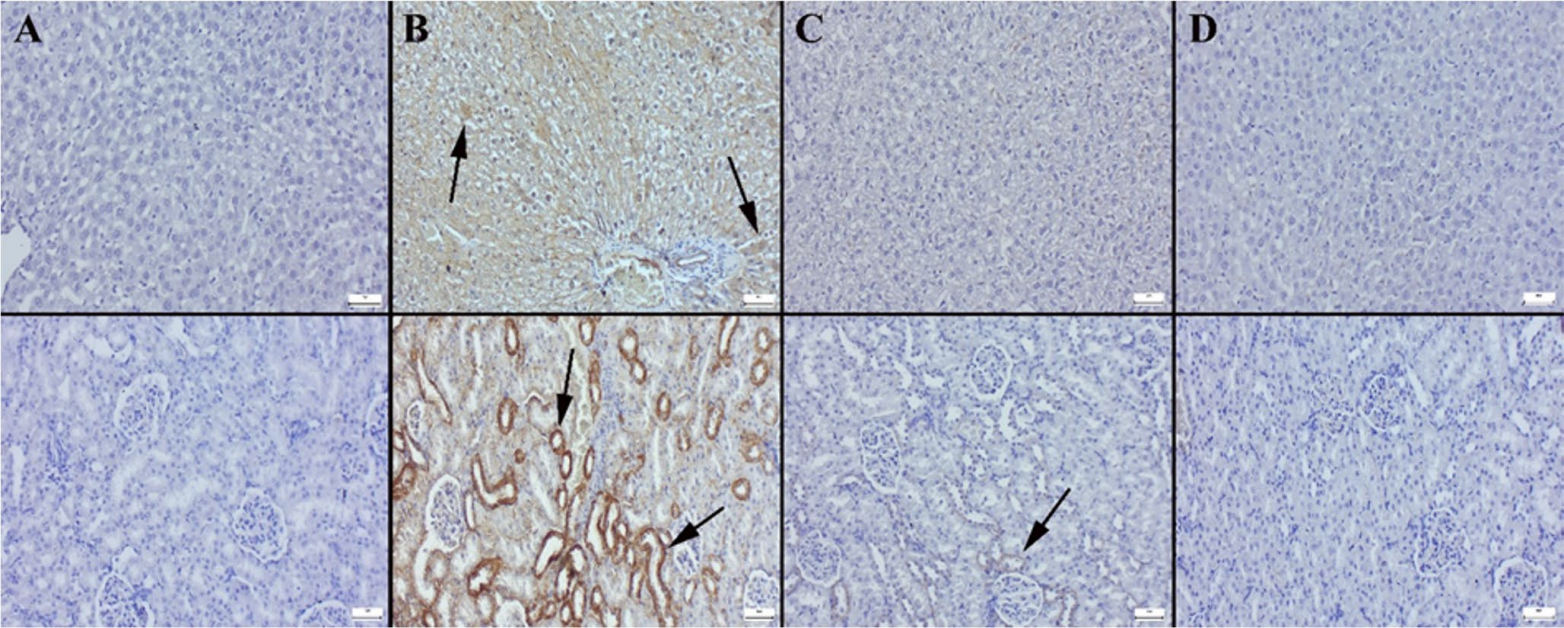
Immunohistochemical localization of caspase-9 expression in liver (upper row) and kidney (lower row) tissues from the experimental groups. (**A**) Control group showing negative caspase-9 immunoreactivity. (**B**) AFB_1_ group demonstrating markedly increased caspase-9 expression in both hepatic and renal tissues (arrows). (**C**) AFB_1_+Ch group showing a pronounced reduction in caspase-9 immunoreactivity. (**D**) Ch group exhibiting negative caspase-9 expression. Streptavidin–biotin peroxidase method; scale bars = 50 μm and x400. *n* = 10

**Fig. 7:**
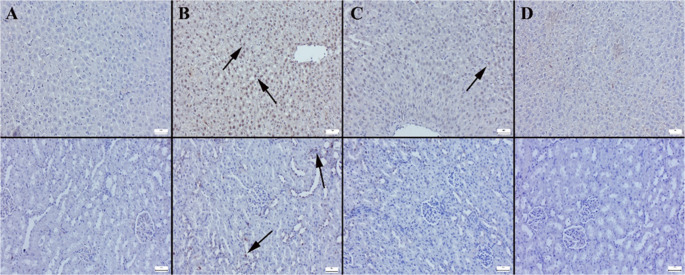
Immunohistochemical localization of Ki-67 expression in liver (upper row) and kidney (lower row) tissues from the experimental groups. (**A**) Control group showing negative Ki-67 immunoreactivity. (**B**) AFB_1_ group exhibiting markedly increased nuclear Ki-67 expression in hepatic and renal cells (arrows). (**C**) AFB_1_+Ch group demonstrating a pronounced reduction in Ki-67 immunoreactivity (arrow). (**D**) Ch group showing negative Ki-67 expression. Streptavidin–biotin peroxidase method; scale bars = 50 μm and x400. *n* = 10

**Fig. 8:**
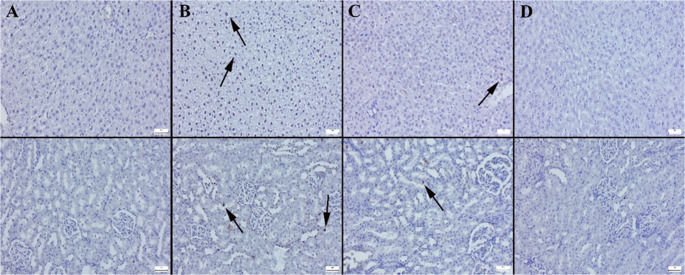
Immunohistochemical localization of PCNA expression in liver (upper row) and kidney (lower row) tissues from the experimental groups. (**A**) Control group showing negative PCNA immunoreactivity. (**B**) AFB_1_ group exhibiting markedly increased nuclear PCNA expression in both hepatic and renal cells (arrows). (**C**) AFB_1_+Ch group demonstrating a pronounced reduction in PCNA immunoreactivity (arrow). (**D**) Ch group showing negative PCNA expression. Streptavidin–biotin peroxidase method; scale bars = 50 μm, and x400. *n* = 10

Immunohistochemical evaluation revealed that AFB_1_-treated animals exhibited a marked increase in the expression of caspase-3, caspase-9, Ki-67, and PCNA in both the liver and kidney tissues. The elevated expression of caspase-3 and caspase-9 was predominantly localized within the cytoplasm of affected cells, indicating enhanced apoptotic activity. In contrast, Ki-67 immunoreactivity was mainly observed in the nuclei, reflecting increased cellular proliferation. Expression of PCNA also showed a clear increase, consistent with heightened DNA synthesis and cell cycle activity. In the control and Ch groups, immunoreactivity for all markers was either negative or very weak, whereas a significant upregulation was observed in the AFB_1_ group. Notably, chrysin treatment markedly reduced the expression levels of caspase-3, caspase-9, Ki-67, and PCNA in the AFB_1_+Ch group, demonstrating its protective effect against AFB1-induced cellular damage. The reduction in immunoreactivity was particularly evident in hepatic hepatocytes and renal tubular epithelial cells, where expression levels approached those observed in the control group. These findings indicate that chrysin effectively attenuates AFB_1_-induced apoptosis and aberrant proliferative responses in both liver and kidney tissues (Figs. 5, 6, 7 and 8).

## Discussion

Aflatoxin B_1_ (AFB_1_) is one of the most potent mycotoxins threatening both human and animal health worldwide, with its toxicity mechanism centered on the disruption of cellular oxidative balance. The reactive electrophilic metabolites produced by the metabolism of AFB_1_ by cytochrome P450 enzymes in the liver covalently bind to macromolecules such as lipids, proteins, and nucleic acids, threatening cellular integrity (Deng et al. 2018; Ma et al. 2021). The functional stability of the cell membrane is closely linked to lipid composition and oxidative status. However, AFB_1_ disrupts lipid metabolism, triggering peroxidation in membrane phospholipids (Rotimi et al. 2016, 2018). This study found that AFB_1_ exposure exacerbated oxidative stress, leading to a dramatic increase in *malondialdehyde* (MDA) levels, a breakdown product of polyunsaturated fatty acids. Excessive MDA production further aggravates the toxic state by forming cross-links with intracellular proteins (Del Rio et al. 2005; Marnett 1999). In contrast, glutathione (GSH), the cell’s most important endogenous defense mechanism, composed of glutamic acid, glycine, and cysteine tripeptide, attempts to limit tissue damage by neutralizing free radicals (Ruffmann and Wendel 1991). In our study, GSH levels were found to be statistically significantly reduced in the AFB_1_-treated group; this indicates that intracellular antioxidant reserves are rapidly depleted to detoxify the excessive ROS load created by the toxin (Zhao et al. 2011). Our current findings are in full agreement with literature data reporting that AFB_1_ increases lipid peroxidation through oxidative damage and suppresses antioxidant capacity (Meki et al. 2001; Ahmed et al. 2022; Altyar et al. 2023; Rotimi et al. 2019). In the chrysin-treated group, the re-elevation of GSH levels and the decrease in MDA levels demonstrate that this flavonoid, as a potent free radical scavenger, restores the depleted defense system and maintains tissue integrity (Khan et al. 2012; Parlak Ak et al. 2021).

The destructive effect of oxidative stress at the cellular level is not limited to cytoplasmic structures but also directly targets the stability of genetic material. Oxidative damage caused by free radicals on DNA leads to strand breaks, base modifications, and DNA-protein cross-linking, initiating the carcinogenesis process (Ercan and Fidancı 2012; Jaruga et al. 2001). 8-OHdG, the most specific biomarker of this damage, is one of the key molecular footprints of AFB_1_ in tumor induction (Shen et al. 1994). The significant increase in 8-OHdG levels detected in the AFB_1_ group in our study confirms the genotoxic potential of the toxin, and this result is consistent with previous studies (Yang et al. 2000; Aleissa et al. 2020; Albadrani et al. 2024). The fact that chrysin application significantly reduced 8-OHdG levels reveals that the compound not only inhibits oxidative stress but also functions as a genoprotective agent that protects nuclear material (Demir et al. 2023; Çomaklı et al. 2023).

This extensive damage to genetic material and membrane structure inevitably activates inflammatory signaling pathways. Oxidative stress triggers the release of pro-inflammatory cytokines such as TNF-α, transforming tissue damage into systemic inflammation (Shen et al. 1994). TNF-α binds to the TNFR1 receptor, stimulating the NFκB and MAPK pathways and exacerbating the inflammatory response (Van Loo and Bertrand 2023). In our study, AFB_1_ exposure was observed to increase TNF-α levels in liver tissue (Wang et al. 2016; Hassaneen et al. 2023), while Chrysin treatment suppressed this increase, exhibiting strong anti-inflammatory efficacy (Li et al. 2010; Yao et al. 2016; Koç et al. 2020; Küçükler et al. 2021). The transcription factor NFκB-p65, which plays a key role in the molecular management of inflammation, regulates genes involved in apoptosis, angiogenesis, and metastasis when activated (Lee and Kim 2009; Friedmann-Morvinski et al. 2016). Our study detected a significant increase in NFκB-p65 levels in liver tissue in the AFB_1_ group (Huang et al. 2019), but no significant difference was found in kidney tissue. This difference in the kidney is thought to be due to the liver’s central role in toxin metabolism, leading to earlier exposure to toxicity, or because the applied dose/duration protocol was below the threshold for activation of this specific pathway in the kidney. Chrysin successfully broke the inflammatory cycle by inhibiting NFκB activation in the liver (Hougee et al. 2005; Yang et al. 2018).

One of the most remarkable and original findings of our study on the molecular regulation of cellular stress response and carcinogenesis is the Sirtuin-1 (Sirt-1) protein. Sirt-1, which has broad effects on gene expression, metabolism, and tumorigenesis (Alcaín and Villalba 2009), plays a paradoxical role in cancer biology as both a tumor suppressor and an oncogenic factor (Bosch-Presegué and Vaquero 2011). While it sometimes inhibits cancer through p53 activation (De Nigris et al. 2002), it has been reported to suppress apoptosis and promote cancer development when overexpressed in many tumor types (Luo et al. 2001; Zhao et al. 2011). In this study, it was determined that Sirt-1 levels in tissues were significantly increased in the group given AFB_1_ compared to the control group. This finding is consistent with Rawat et al. (2020)’s view that high Sirt-1 expression may increase tissue toxicity. The extremely limited number of studies in the literature showing an increase in Sirt-1 as a result of AFB_1_ metabolism makes this finding quite valuable from a scientific perspective. However, the fact that chrysin administration reduces increased Sirt-1 levels differs from studies such as Alanazi et al. (2024) and Lee et al. (2024), which argue that chrysin activates Sirt-1. This difference suggests that in our model, chrysin alleviates hepatorenal damage by suppressing oncogenic signals through a different molecular pathway and reducing cellular stress (thus eliminating the need for compensatory or pathological Sirt-1 increase).

Another critical and unique parameter in assessing the carcinogenic process and metastasis risk is sialic acid. Sialic acid, located at the terminal ends of cell surface glycoconjugates, plays a key role in cellular recognition and communication (Schauer and Kamerling 2018). Increased sialic acid levels and abnormal sialoglycan expression show a strong correlation with malignant transformation, tumor growth, and metastasis processes (Hekim 2008; Öksüz et al. 2020; Visser et al. 2021). In this study, serum sialic acid levels were found to be significantly elevated in the AFB_1_-induced hepatorenal damage model. This result is consistent with studies reporting increases in different cancer and disease models (Cemek et al. 2010; Hodgson et al. 2024). However, a literature search revealed no other studies examining changes in sialic acid levels in AFB_1_ toxicity. Given the limited data suggesting that flavonoids can control sialic acid expression (Hidari et al. 2009; Yoshinaga et al. 2016), our study found that Chrysin administration reduced increased serum sialic acid levels. This finding can be considered one of the first in the literature to demonstrate Chrysin’s suppressive effect on metastatic potential in vivo.

All these damages occurring at the molecular and biochemical levels are concretely reflected in organ functions and tissue architecture. It is known that serum AST, ALT, and GGT activities increase in liver damage (Jayabalan et al. 2010), while creatinine and urea levels increase in kidney damage (Kamal 2014). In our study, the significant increases observed in liver enzymes in the AFB_1_ group were consistent with the literature (Abdel-Daim et al. 2021); however, no statistically significant difference was found in kidney function tests (creatinine, BUN). Although this contradicts some previous studies (Rotimi et al. 2018; Hatipoğlu and Keskin 2022), it can be explained by the dose- and duration-dependent nature of toxicity (Aleissa et al. 2020) and the fact that the 7-day protocol we applied was an early phase for the reflection of kidney damage. Histopathological studies showing that Chrysin improves necrotic and degenerative changes in liver and kidney tissue (Zhang et al. 2004; Fan et al. 2021) morphologically support our biochemical findings.

Finally, the cellular response to tissue damage was evaluated through the balance of apoptosis and proliferation. It was determined that AFB_1_ induces apoptosis by increasing the expression of Caspase-3 and Caspase-9 through mitochondrial damage (Liu and Wang 2016), and also increases the levels of PCNA (Kelman 1997; Qin et al. 2016), a marker of necrosis and cell proliferation, and Ki-67, a tumor proliferation marker (Scholzen and Gerdes 2000; Uxa et al. 2021). The absence of studies in the literature examining the relationship between AFB_1_ and Ki-67 increases the significance of our findings. The simultaneous increase in both apoptotic (caspases) and proliferative (PCNA, Ki-67) markers in the AFB_1_ group reflects tissue destruction and disordered regeneration efforts. Chrysin treatment restored tissue homeostasis by reducing the expression of these apoptotic and proliferative factors (Woo et al. 2004; Coşkun et al. 2021 Gür et al. 2022), and inhibited carcinogenic progression at the molecular level via critical pathways such as Sirt-1 and sialic acid.

## Conclusion

According to these research findings, AFB_1_ administered at a dose of 0.5 mg/kg/BW was found to cause lipid peroxidation, decreased antioxidant defense system activity, increased cytokine levels, and increased inflammatory enzyme activity as a result of ROS-induced tissue damage. Furthermore, AFB_1_ was found to cause immunohistochemical and histopathological changes (increased expression of caspase-3, caspase-9, Ki-67, and PCNA). On the other hand, it was determined that chrysin administration at 25 mg/kg/BW prevented the biochemical and histopathological changes caused by AFB_1_. In conclusion, chrysin can be used as an antioxidant, anti-inflammatory, and anticancer agent against AFB_1_-induced liver and kidney damage.

## Data Availability

The data that support the findings of this study are available from the corresponding author upon reasonable request.

## Abbreviations

AF: Aflatoxin
AFB_1_: Aflatoxin B_1_
Ch: Chrysin
Sirt-1: Sirtuin 1
GGT: Gamma-glutamyl transferase
ALT: Alanine aminotransferase
AST: Aspartate aminotransferase
CAT: Catalase
GSH: Glutathione
GST: Glutathione S-transferase
MDA: Malondialdehyde
SOD: Superoxide dismutase
TNF-α: Tumour necrosis factorα
ROS: Reactive oxygen species
CYP450: Cytochrome P450
8-OHdG: 8-hydroxydeoxyguanosine
NFκB-p65: Nuclear factor kappa B-p65
BUN: Blood urea nitrogen
